# Supplementary figures and images for: Changes in long non-coding RNA transcriptomic profiles after ischemia-reperfusion injury in rat spinal cord
Source: PeerJ. 2020 Jan 6;8:e8293. doi: 10.7717/peerj.8293 (PMC6951290; doi:10.7717/peerj.8293)

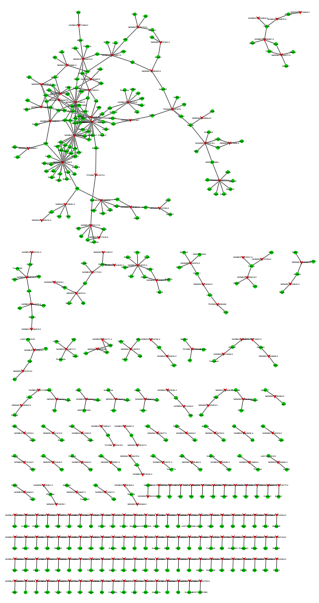

Supplement: Figure S1 — Arrows represent DElncRNAs, and ellipse nodes represent DEmRNAs. [file peerj-08-8293-s001.pdf]
